# A new structure-property connection in the skeletal elements of the marine sponge *Tethya aurantia* that guards against buckling instability

**DOI:** 10.1038/srep39547

**Published:** 2017-01-04

**Authors:** Michael A. Monn, Haneesh Kesari

**Affiliations:** 1 School of Engineering, Brown University, Providence, RI, USA

## Abstract

We identify a new structure-property connection in the skeletal elements of the marine sponge *Tethya aurantia*. The skeletal elements, known as spicules, are millimeter-long, axisymmetric, silica rods that are tapered along their lengths. Mechanical designs in other structural biomaterials, such as nacre and bone, have been studied primarily for their benefits to toughness properties. The structure-property connection we identify, however, falls in the entirely new category of buckling resistance. We use computational mechanics calculations and information about the spicules’ arrangement within the sponge to develop a structural mechanics model for the spicules. We use our structural mechanics model along with measurements of the spicules’ shape to estimate the load they can transmit before buckling. Compared to a cylinder with the same length and volume, we predict that the spicules’ shape enhances this critical load by up to 30%. We also find that the spicules’ shape is close to the shape of the column that is optimized to transmit the largest load before buckling. In man-made structures, many strategies are used to prevent buckling. We find, however, that the spicules use a completely new strategy. We hope our discussion will generate a greater appreciation for nature’s ability to produce beneficial designs.

Biological materials often possess quite distinct mechanical designs. The designs range from the overall shape of biological structures at the large-scale, to intricate 3D architectural motifs at the small-scale^1^,^2^. The shape of scales and claws^3^,^4^, the truss-like internal structure of vulture wings^5^, the brick-and-mortar arrangement of mineral tablets in mollusc shells^6^, and the graded porosity of grass stems^7^ demonstrate the diversity and visually striking nature of these mechanical designs. Some of these mechanical designs are products of unyielding evolutionary pressures and are believed to enhance the properties of their corresponding structures and materials. Consequently, new strategies for improving a structure’s or a material’s performance can be discovered by studying the structure-property connections in these mechanical designs. For example, structure-property investigations motivated by the remarkable toughness of nacre and bone have led to the development of new bio-inspired structural ceramics^8^,^9^,^10^.

Structure-property investigations have primarily focused on toughness-related mechanical properties for the past forty years^6^,^11^,^12^. Only a small amount of attention has been devoted to other equally important mechanical properties, such as strength, stiffness, and buckling resistance^13^,^14^,^15^. Buckling is the phenomenon in which a slender, structural element that is subjected to an increasing axial compressive force abruptly starts to deform laterally when the force’s magnitude reaches a critical value. This instability dramatically reduces the structure’s ability to provide stiffness and structural support, and in many cases can lead to catastrophic failure.

There has always been a need for buckling-resistant designs at the large-scale, e.g., in light-weight aerospace and civil engineering structures^16^,^17^. Recently, however, understanding and controlling buckling has also become important at the small-scale as well. A number of stretchable electronics platforms being developed are based on the design of micro-scale structures whose buckling instabilities can be precisely controlled^18^,^19^,^20^. Bio-medical instruments, such as needles, catheter guidewires, and stents depend on buckling resistance in order to effectively penetrate tissue or be inserted through narrow ducts or capillaries^21^,^22^,^23^. Stents must also provide reliable and long-term mechanical support to the surrounding tissue^22^,^23^.

We identify a new connection between the mechanical design and buckling resistance in the skeletal elements of the marine sponge *Tethya aurantia. T. aurantia* is a sessile animal that grows on rocky surfaces in the Mediterranean^24^. The skeletal elements that we focus on are needle-shaped structures called strongyloxea spicules (see Fig. 1). The strongyloxea (Sxa) are monolithic, axially symmetric, silica rods (see Fig. 2(b)). They are roughly 35 *μ*m thick, 2 mm long, and are tapered along their length (see Supplementary Section *Details of Sxa profile measurements* and Fig. 1(a)). We found that the tapered shape is remarkably uniform across different Sxa (see Section *Measurement of Sxa profiles* and Fig. 1(d)). Considering that sponges have a great degree of control over the shape of their spicules, it is natural to wonder whether this tapered shape has some functional significance.

We introduce and investigate the hypothesis that the Sxa’s taper is an adaptation aimed at enhancing their ability to provide stiffness to the sponge. Our hypothesis is motivated by the following observations. (a) *Mechanical stiffness is important for the sponge. T. aurantia* is primarily found in shallow, coastal environments, where it is subjected to forces exerted by underwater waves and currents^24^,^25^,^26^. It feeds by filtering microscopic organic particles and microorganisms from seawater. Large deformations of the sponge’s body caused by ambient loads could inhibit its ability to feed. Therefore, it is critical that the sponge’s body be stiff enough to limit any such large deformations. (b) *The sponge derives its stiffness primarily from the Sxa*. The Sxa are distributed within the sponge’s spherical body and are embedded in a collagenous matrix, called spongin (see Fig. 3(a))^26^,^27^. Spongin is very compliant, having a Young’s modulus of only 600 KPa^28^. The Sxa on the other hand are composed of silica, which has a Young’s modulus of 72 GPa^29^. The Sxa also lack any internal structure (see Fig. 2(b)) that would imply that they perform functions other than to provide mechanical support to the sponge. Finally, a closely related sponge—*Tethya citrina*—that grows in calmer waters is more compliant and produces fewer spicules per body volume^26^. This is consistent with mechanical tests performed on spicule containing tissues, which show that the spicules drastically increase the tissue’s stiffness^30^. (c) *The Sxa’s ability to provide stiffness is limited by their resistance to buckling*. (d) *The buckling resistance of a slender structure can be increased by tapering it*. The destabilizing bending moments arising from the eccentricity of a structure’s axial compressive loads are more intense at the structure’s center than at its ends. Hence, its buckling resistance can be enhanced by moving material away from its ends, towards its center. This result has been established both theoretically^31^ and experimentally^32^. We elaborate on this result further in Section *Comparison with the Clausen profile*.

We test our hypothesis as follows. Based on mechanical testing (see Section *Mechanical testing of Sxa*) and sponge-anatomy informed computational mechanics calculations (see Section *Computational mechanics calculations* and Supplementary Section *Computational mechanics model of a Sxa in its RoC*), we construct a structural mechanics model for the Sxa (see Section *The structural mechanics model for the Sxa*). Using our model, we identify the shape of the structure that has the greatest resistance to buckling (see Section *Comparison with the Clausen profile*). Finally, we measure the Sxas’ tapers from SEM images and compare them with the shape of this optimal structure (see Section *Comparison with the Clausen profile*). We find that the Sxas’ tapers are strikingly similar to the shape of the optimal structure. This similarity suggests that the Sxas’ tapered shape enhances their resistance to buckling.

Our mechanical tests are discussed in Section *Mechanical testing of Sxa*. They show that the Sxa behave in a linear elastic fashion until failure. They also show that the Sxa’s deformation behavior in bending can be modeled exceptionally well using classical structural mechanics theories (see Fig. 2(d)). Furthermore, from the Sxa’s arrangement within the sponge’s body it is clear that the Sxa’s primary function is to stiffen the sponge against radial compressive stresses^26^,^27^,^30^. We analyze a Sxa and a small section of its surrounding spongin matrix using computational mechanics calculations that are consistent with the sponge’s skeletal anatomy (see Section *Computational mechanics calculations* and Supplementary Section *Computational mechanics model of a Sxa in its RoC*). The results from our computational mechanics calculations show that due to the difference in the stiffnesses of the Sxa and spongin, the spongin matrix transmits the radial compressive stresses to the Sxa as highly localized surface tractions on their ends (see Supplementary Section *Computational mechanics model of a Sxa in its RoC*). Synthesizing the knowledge gained from the mechanical tests, and the computational mechanics simulations, we model the Sxa as a simply supported column (see Section *The structural mechanics model for the Sxa*).

In the column model, the Sxa’s stiffening ability is limited by the Euler buckling instability. Thus, the Sxa’s stiffening ability can be quantified by what we call its buckling strength, which is the maximum axial compressive force that it can transmit without buckling. The shape that would be most consistent with our hypothesis would be the one for which the column model attains its maximum buckling strength. It has been shown using rigorous mathematical techniques that the buckling strength of a simply-supported column can be enhanced by up to 33% over that of a cylinder by tapering it so that its radius as a function of length is described by what we call the Clausen profile^33^,^34^. Thus, to test our hypothesis we check how well the Sxa’s tapered shape is described by the Clausen profile.

We imaged 31 Sxa using scanning electron microscopy (SEM) and measured their profiles. In order to interpret how well the measured profiles compare with the Clausen profile, we compare them to not only the Clausen profile but also to other prototypical tapered profiles (see Section *Comparison with the Clausen profile*). By fitting the profile models to the measured profiles, we find that the Clausen profile describes the Sxa’s tapered shape the best (see Fig. 4(B)).

We do not directly measure the buckling strengths of the spicules. However, we use our measurements of the Sxas’ profiles along with our structural mechanics model to estimate the buckling strengths of the Sxa (see Section *Direct estimates of the Sxas*’ *buckling strengths*). We compare the estimated buckling strengths of the Sxa to the buckling strengths of equivalent cylinders—i.e., cylinders with the same length, volume, and elastic properties (see Fig. 5). We find that the buckling strengths of the Sxa predicted by our model can be as much as 30% greater than those of their equivalent cylinders. This is close to the 33% enhancement that is achieved by the Clausen profile.

The resemblance of the Sxa’s profile to the Clausen profile is quite striking and supports our hypothesis. However, our work is only a first step in understanding the functional significance of the Sxa’s tapered shape. It is possible that the Sxas’ tapered shape serves a mechanical function that is different from the one that we have presumed. Or, it is also possible that the taper is simply a consequence of the spicular growth processes, and its resemblance to the Clausen profile is only a misleading coincidence. These possibilities cannot be ruled out without having more information about the sponge’s anatomy and ethology. The most direct way to reject our hypothesis would be to show that at least one of our key assumptions is incorrect. These key assumptions pertain to: (i) the importance of stiffness to the sponge, (ii) the primary function of the Sxa, (iii) the role of the buckling instability in dictating the Sxa’s stiffening ability, and (iv) the effect of the spongin matrix on the Sxa’s buckling behavior.

## Results

### Measurement of Sxa profiles

**Figure 1:**
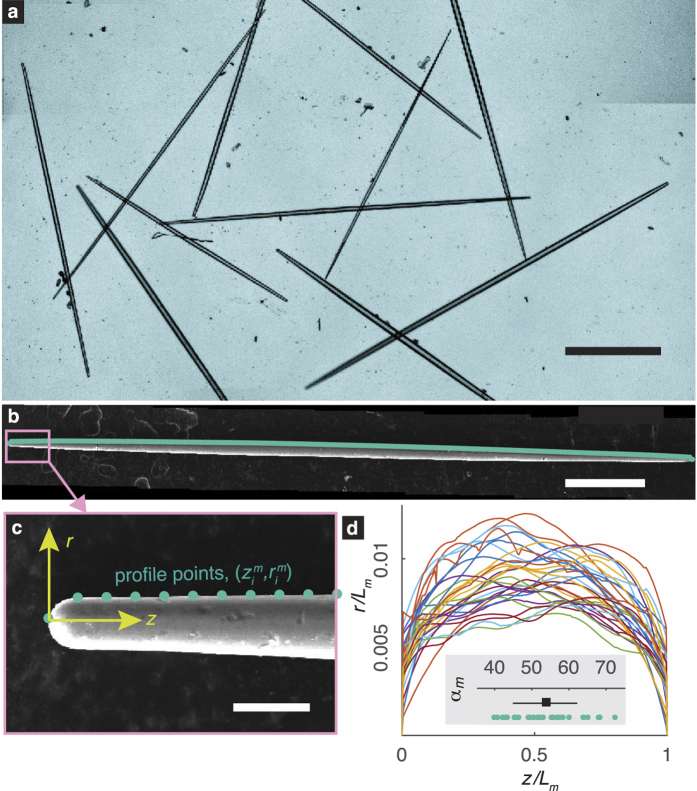
Measurement of Sxa profiles. (**a**) A micrograph of several Sxa. (**b**) An SEM image of a single Sxa. The Sxa’s profile is highlighted. (**c**) A magnified view of (**b**) showing points composing the profile. (**d**) Dimensionless profiles of the 31 Sxa. The inset shows the distribution of *α*_*m*_. The square and error bar indicate the mean and standard deviation of *α*_*m*_. The scale bars in (**a**)–(**c**) are 500 *μ*m, 250 *μ*m and 25 *μ*m, respectively.

We extracted the shape of 31 Sxa from SEM images (see *Methods*). Since the Sxa are axisymmetric, we describe a Sxa’s shape using its “profile”, which is a set of points 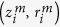, *i* = 1 … 250 shown in Fig. 1(b,c). We measured the length, *L*_*m*_, and maximum cross-sectional radius, *R*_*m*_, of each Sxa from its profile (see Supplementary Section *Details of Sxa profile measurements*). By plotting the dimensionless profiles, 
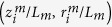
, for *i* = 1 … 250, we see that the general nature of the taper appears uniform across different Sxa. To make a more quantitative comparison of the Sxas’ tapers we compute the aspect ratio, *α*_*m*_ = *L*_*m*_/2*R*_*m*_, for each Sxa. The values of *α*_*m*_ are plotted in Fig. 1(d). The mean and standard deviation of *α*_*m*_ are 53.6 and 8.7, respectively. The small scatter of *α*_*m*_ further supports our viewpoint that the tapered shape is uniform across different Sxa.

### Mechanical testing of Sxa

**Figure 2:**
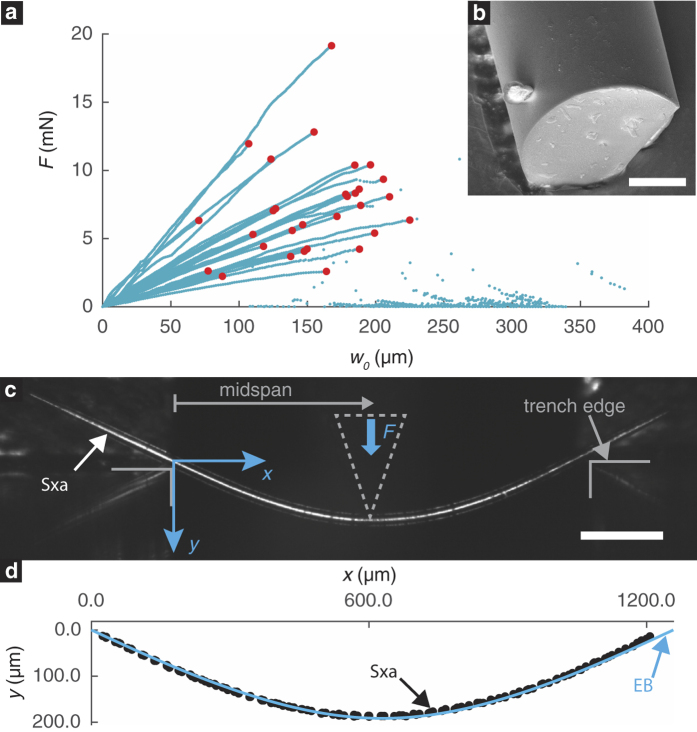
Three-point bending tests of Sxa. (**a**) Applied force, *F*, versus displacement at midspan, *w*_0_, for 30 Sxa. Red points indicate the load and displacement at which each Sxa failed. (**b**) A cross-section of a fractured Sxa. (**c**) Micrograph of a bent Sxa just prior to failure. The indenter used to apply the force is outlined with dashed lines. (**d**) Points along the Sxa’s axis are obtained from (**e**). The blue curve labeled EB is the deformed shape predicted by Euler-Bernoulli theory. The scale bars in (**b**) and (**c**) are 10 *μ*m and 250 *μ*m, respectively.

The Sxa are primarily composed of silica^35^, which is a well-characterized ceramic material that behaves in a linear elastic fashion and fails through brittle fracture. Cursory inspection of the surfaces of fractured Sxa (see Fig. 2(b)) suggests that they are essentially homogeneous silica rods. However, spicules also possess a proteinaceous scaffold within their silica^36^,^37^. In some related species this protein forms distinct layers, which may affect the deformation and failure behavior of the spicules^13^,^35^,^38^,^39^. While *T. aurantia*’s Sxa do not contain separate layers of protein and silica, the influence of any underlying protein scaffold on their elastic behavior is unknown. Furthermore, the composition of the silica itself varies spatially within the Sxa^40^. To ascertain the effect of any potential elastic inhomogeneities on a Sxa’s deformation behavior, we performed three-point bending tests on 30 Sxa using a custom-built flexure device. Briefly, the Sxa were suspended over a trench with vertical, parallel walls and were indented by a cantilever that also acts as a force sensor. The details of our flexure device and three-point bending tests will be published elsewhere.

The magnitude of the transverse force, *F*, and deflection of the Sxa’s axis in the *y*-direction at midspan, *w*_0_ (see Fig. 2(c)), were recorded until the Sxa failed. The *w*_0_–*F* data for the Sxa are shown in Fig. 2(a). The failure of every Sxa we tested was defined by a single fracture event. The fracture events are marked with red points in Fig. 2(a). The *w*_0_–*F* response of every Sxa was linear until failure. This observation indicates that the Sxa’s mechanical behavior is linear elastic until failure.

We compared a Sxa’s deformed shape during a bending test to that predicted by Euler-Bernoulli theory for an elastically homogeneous, tapered beam^41^. Euler-Bernoulli (EB) theory is a highly successful structural mechanics theory used for modeling the deformation of slender, linear elastic structures that primarily deform through bending. For details of how the shape predicted by EB theory was computed, see Supplementary Section *Deflection of a tapered beam in three*-*point bending predicted by Euler*-*Bernoulli theory*. The displacement of a Sxa’s axis in the *y*-direction was measured from images taken during the bending test (see Fig. 2(c)). A representative comparison of these measured displacements with those predicted by EB theory is shown in Fig. 2(d). The measurements and the theoretical predictions match very well for 27 of the 30 Sxa. This supports that the Sxa’s behavior is linear elastic and shows that a Sxa is elastically homogeneous along its length. Furthermore, it shows that a Sxa’s deformation can be described by an EB theory for an elastically homogeneous, tapered, axially symmetric beam.

### Computational mechanics calculations

Being embedded within the sponge, a Sxa likely experiences a complex distribution of tractions along its length. However, using computational mechanics calculations we found that due to the Sxas’ arrangement within the sponge and the large mismatch between the compliance of the Sxa and the spongin, the tractions are localized at the ends of the Sxa (see Supplementary Section *Computational mechanics model of a Sxa in its RoC*). Thus, the most appropriate structural mechanics model based on EB theory would be a simply supported column, which is described by equations (1,2,3).

**Figure 3:**
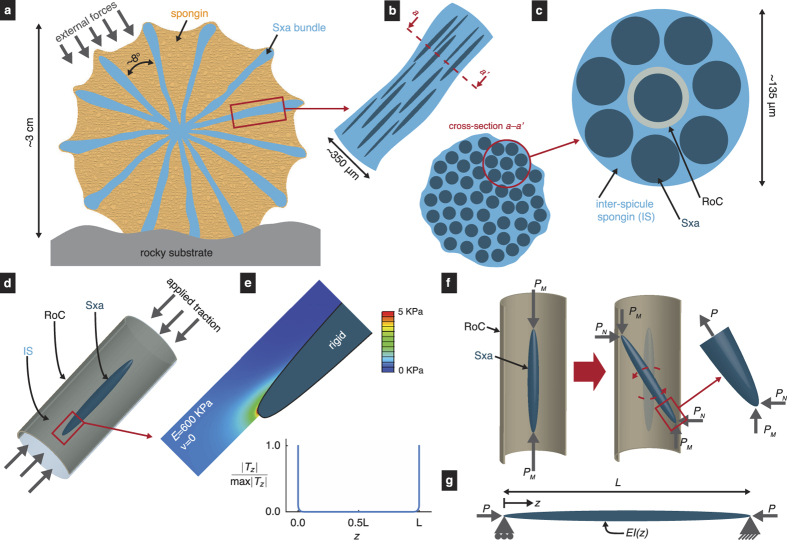
Arrangement of Sxa within the sponge motivates a structural mechanics model. (**a**) A cross-section of the sponge reveals radial bundles of Sxa. (**b**) A bundle is composed of Sxa (dark) separated by spongin (light). (**c**) The presence of neighbors limits the deformation of a Sxa to a region of confinement (RoC). (**d**) Tractions applied to the ends of the RoC are transferred to the Sxa by the inter-spicule spongin (IS). (**e**) Von Mises stress computed from a computational mechanics model of (**d**). The distribution of axial force per unit length, *T*_*z*_, along the length of a Sxa is localized at the ends. (**f**) A Sxa within its RoC, subjected to opposing forces with magnitude *P*_*M*_ applied at its ends. A Sxa rotates until it is restrained by the presence of neighboring Sxa. The net force acting along a Sxa’s axis has a magnitude *P*, which includes contributions from *P*_*M*_ and *P*_*N*_. The Sxa and RoC in (**d**)–(**f**) are not to scale. (**g**) A schematic of a simply supported column.

The Sxa are not uniformly scattered throughout the sponge’s body, rather they are grouped in bundles that extend radially from the sponge’s center to its outer surface (see Fig. 3(a))^26^. The Sxa are aligned along the bundles’ lengths and are staggered with respect to each other (see Fig. 3(b)). The bundles are 220–490 *μ*m thick^26^ and a bundle’s cross-section contains approximately 50 Sxa^27^. From the average bundle thickness, number of Sxa per bundle, and Sxa diameter, we estimate the distance between the axes of neighboring Sxa to be ≈45 *μ*m (see Supplementary Section *Estimation of the distance between adjacent Sxa in a bundle*). Thus, the Sxa within a bundle are separated from each other by a small amount, ≈8 *μ*m, of spongin.

External forces acting on the sponge are transmitted by the spongin to the Sxa as tractions on their surfaces. To determine the distribution of these tractions, we performed a stress analysis on a continuum mechanics model of an individual Sxa embedded in a cylindrical section of spongin. We refer to this cylinder as a Sxa’s region of confinement (RoC) (see Fig. 3(c,d)). The diameter of the RoC is equal to the distance between neighboring Sxa in a bundle.

We model the spongin in the RoC as an isotropic, linear elastic solid with Young’s modulus and Poisson’s ratio of 600 KPa and 0, respectively. These values correspond to measurements of the mechanical properties of spongin in a related species^28^. Furthermore, measurements of the Young’s modulus of spicules from a related species^42^ indicate that the silica is between four and five orders of magnitude stiffer than the spongin. We will present Young’s modulus measurements of the Sxa from our own work in a future paper. Motivated by this large difference in stiffnesses, we model a Sxa as a rigid inclusion whose surface is bonded to the spongin in its RoC. We assume that external forces act normal to the sponge’s surface and result in axial compressive stresses in the Sxa bundles. Therefore, we apply compressive tractions to the ends of the RoC (see Fig. 3(d)). Since the spongin in a RoC is also connected to the spongin in the RoCs of neighboring Sxa, we constrain points on the lateral surface of the RoC from moving in the radial direction. Further details about this model can be found in Supplementary Section *Computational mechanics model of a Sxa in its RoC*.

We computed the stress field in the spongin using finite element procedures (see Fig. 3(e))^43^. We found that for a wide range of traction distributions applied to the ends of the RoC, the axial force per unit length acting on the Sxa is always localized on the Sxa’s ends (see Fig. 3(e) and Supplementary Section *Computational mechanics model of a Sxa in its RoC*). This localized force distribution contrasts with that predicted for an ellipsoidal inclusion embedded in a linear elastic solid subjected to far-field compressive stress. Specifically, a celebrated elasticity solution by Eshelby^44^ predicts that the axial force per unit length will vary in a piecewise affine fashion along an ellipsoidal inclusion. It is not necessary, however, for this result to hold true for non-ellipsoidal inclusions. Thus, our numerical results do not contradict Eshelby’s solution. In fact, they are consistent with results from computational models of short fiber reinforced composites^45^,^46^, full-field elasticity solutions for rigid line inclusions^47^, and photoelasticity experiments on line-like inclusions^48^. Based on the insight gained from our computational mechanics calculations, we modeled the effect of the spongin by replacing the tractions applied to the ends of the RoC with opposing point forces, ±**P**_**M**_ at the Sxa’s ends (see Fig. 3(f)).

### The structural mechanics model for the Sxa

Initially a Sxa behaves like a column with two free ends, which is unstable when subjected to the the axial forces ±**P**_**M**_. Even if these forces are aligned with the Sxa’s axis, small perturbations in the configuration will inevitably cause the Sxa to rotate about one of its transverse axes. However, after rotating by only a small amount (≈1.3°), the proximity of neighboring Sxa in the bundle will prevent further rotation (see Fig. 3(f)). Due to the spongin’s large compliance, it is unlikely that this small rotation will substantially change the stress state in the spongin and consequently the traction distribution on the Sxa’s surface. However, there will be non-negligible reaction forces, ±**P**_**N**_, at the points where a Sxa is restrained by its neighbors. The net force at a Sxa’s end, **P**, which includes contributions from **P**_**M**_ and **P**_**N**_, must act in the direction of the Sxa’s axis (see Fig. 3(f)). This is a consequence of static equilibrium and can be deduced using a free body diagram.

Thus, a Sxa can be modeled using the EB theory in which the column’s ends are subjected to compressive, axial forces and cannot move in the direction perpendicular to the column’s axis. We refer to this model as a simply supported column (see Fig. 3(g)). In this model, the transverse deflection, *w*, is governed by the differential equation


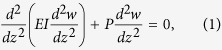


for all *z* ∈ (0, *L*), and boundary conditions


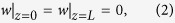



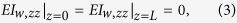


where *P, E, L* and *I* are the magnitude of **P**, the column’s Young’s modulus, length and second moment of area, respectively. Based on the results of Sections *Measurement of Sxa profiles* and *Mechanical testing of Sxa* we take *E* to be constant and *I*(*z*) = *πr*(*z*)^4^/4, where *r*(*z*) is the radius of the Sxa’s cross-section—i.e. its profile.

### Comparison with the Clausen profile

The buckling strength of a simply supported column is the smallest *P* for which there exists a solution to equations (1,2,3) other than *w* = 0 for all *z* ∈ [0, *L*]. For an elastically homogeneous column, the buckling strength can be modulated by varying *I*, or in this case *r*, along the column’s length^31^. Our hypothesis would gain support if the profile of the simply supported column with the greatest buckling strength resembled the measured profiles of the Sxa.

**Figure 4:**
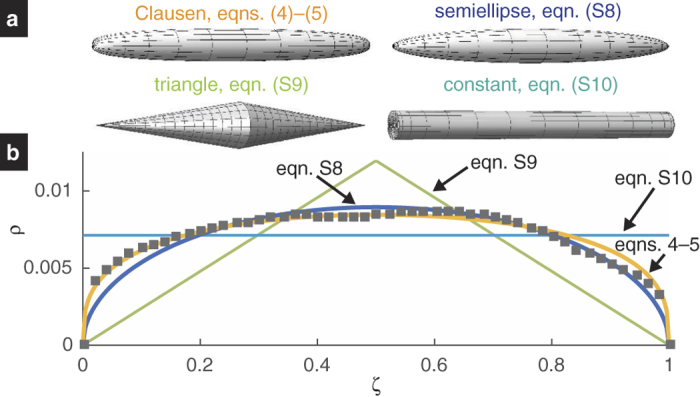
Comparison of a Sxa’s taper to several profiles. (**A**) Columns whose profiles are given by equations (4) and (5) and (S8–S10). (**B**) The best fit profiles for a representative Sxa. The dimensionless Sxa profile points are shown as gray squares.

The profile that maximizes a simply supported column’s buckling strength for a given length, *L*, and volume, *V*, was first sought by Lagrange in the late 1700s^49^. The correct solution, however, was discovered in 1851^50^, and an accessible proof that it is in fact optimal was given in 1962^34^. This optimal profile, which we refer to as the Clausen profile, is given by


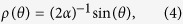



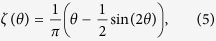


where *ρ* = *r*/*L* and *ζ* = *z*/*L* are the dimensionless radial and axial coordinates, respectively, and *θ* is a parameter that lies between 0 and *π*^33^,^34^. The parameter *α* = (3*πL*^3^/16*V*)^1/2^ is a measure of the column’s aspect ratio. We refer to a column whose taper is described by the Clausen profile as a Clausen column (see Fig. 4(A)).

To test our hypothesis, we compared the Clausen profile to the Sxa profiles. We did this by fitting equations (4) and (5) to each Sxa profile in the least-squares sense by varying the parameter *α* (see Supplementary Section *Fitting profiles to the Sxas*’ *shape*). The best fit Clausen profile for a representative Sxa is shown in Fig. 4(B). We also fit three other prototypical profiles; a semiellipse, an isosceles triangle and a constant to the Sxa profiles (see Supplementary Section *Fitting profiles to the Sxas*’ *shape* and Fig. 4(A)). We use the sum of squared residuals for a fitted profile, *mSSR*, to indicate how well that profile describes a Sxa’s shape. The medians, means and standard deviations of each profile’s *mSSR* are shown in Table 1, from which we see that the Clausen profile has the lowest mean and median *mSSR*. Furthermore, a two-sided Wilcoxon signed rank test indicates that the median *mSSR* for the Clausen profile differs from that of the semiellipse profile at the 1% significance level (*p* = 0.0002). Thus, using the median *mSSR* as a metric, we conclude that the Clausen profile describes the Sxas’ tapers the best out of the different profiles that we considered. Further investigation using another comparison criterion also supports this conclusion (see Supplementary Section *Additional profile comparison using the Akaike information criterion* for details).

### Direct estimates of the Sxas’ buckling strengths

The fact that the Clausen profile describes the measured Sxa profiles the best among the prototypical tapered profiles that we considered gives strength to our hypothesis. However, it is still possible that there may exist some other profile, which corresponds to an alternate hypothesis, that describes the Sxa’s taper even better than the Clausen profile. If such a profile exists, would our hypothesis remain viable?

**Figure 5:**
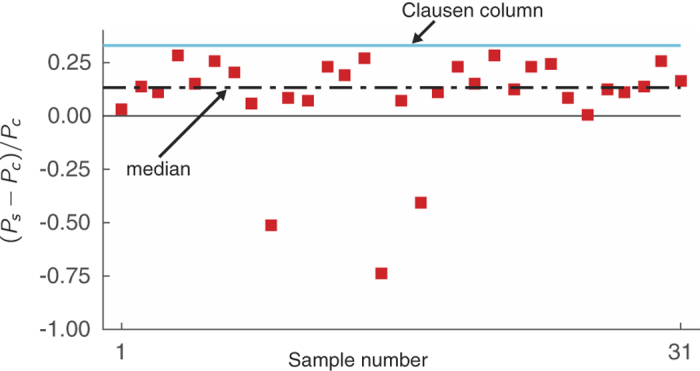
Estimated buckling strengths of Sxa. The relative buckling strengths, (*P*_*s*_ − *P*_*c*_)/*P*_*c*_, of the 31 Sxa whose profiles were measured in Section *Measurement of Sxa profiles* are estimated using our structural mechanics model an are shown as red squares. The dashed, black line indicates the median of the Sxas’ relative buckling strengths. The solid, blue line denotes the maximum possible enhancement of buckling strength, which corresponds to the Clausen column.

To answer this question, we numerically estimated the Sxas’ buckling strengths, *P*_*s*_, using the measured profiles and our structural mechanics model. Briefly, we computed a Sxa’s second moment of area *I*(*z*) = *πr*(*z*)^4^/4 from its profile, *r*(*z*), and used the Rayleigh-Ritz method^51^ to find an approximate value for the smallest *P* for which there exists a solution to equations (1,2,3) other than *w* = 0. We computed *P*_*s*_ for each of the 31 Sxa whose profiles we measured in Section *Measurement of Sxa profiles* and compared it to the buckling strength *P*_*c*_ = *πEV*^2^/(4*L*^4^) of the equivalent cylinder—i.e., the cylinder with the same length, volume, and elastic properties (see Fig. 5). Taken as a group, we found that the median buckling strength of the Sxa is 13.4% greater than that of their equivalent cylinders. Furthermore, some Sxa achieve values of (*P*_*s*_ − *P*_*c*_)/*P*_*c*_ as large as 0.3 which is close to the enhancement of 0.33 provided by the Clausen column^33^ (see Fig. 5).

So, even if there existed a profile that better resembled the Sxas’ tapers, the fact still remains that the Sxas’ tapers substantially enhance their buckling strengths. Therefore, even if there existed a better matching profile based on an alternate hypothesis, the support for our hypothesis would still remain strong. Such a scenario would only mean that the Sxa serve more than one function.

## Discussion and Concluding remarks

The structure-property connection that we identify in the Sxa represents a completely new type of entry into the growing library of structure-property connections in biological materials and structures. This new structure-property connection is related to buckling resistance rather than toughness enhancement, which is the focus of the majority of past structure-property investigations. While the identified connection is related to the structure’s stiffness, by being sharply focused on preventing buckling it is quite different from the stiffness-related structure-property connections that have been identified in biological structures, such as stems and quills^14^,^15^. We hope that our work encourages the investigation of the potential buckling resistance offered by the tapered shapes of other slender biological structures, such as hedgehog quills and echinoderm spines.

Though the result that tapering a slender structure can increase its buckling strength is well known in the applied mathematics community, it has not been widely adopted by the engineering community for the design of buckling-resistant structures. The Sxa demonstrate that tapering structures to increase their buckling resistance is indeed useful in practice. It would be interesting to see how engineers extrapolate this result to more general structures, such as trusses. Quantifying the enhancements in such generalizations will lead to the formulation of some very interesting mathematics and mechanics problems.

We also believe that this work will increase the interest in structure-property investigations. Interest in bio-inspired engineering was originally based on the tacit assumption that evolutionary adaptation produced close-to-optimal mechanical designs^52^. However, now it is understood that for adaptations to take root they do not have to be close-to-optimal, but only “good enough”^53^. This understanding stemmed from the fact that there are very few examples of mechanical designs in biological structures and materials that have been rigorously shown to be close-to-optimal^13^,^54^,^55^,^56^. This new understanding acts as an important bulwark against efforts that blindly imitate mechanical designs in biology without first understanding their functional significance. Unfortunately, this new understanding can also lead to excessive skepticism about the effectiveness of adaptations, and consequently, about the importance of investigating structure-property connections. Since our results show that the taper in Sxa is not just a beneficial adaptation, but is in fact a close-to-optimal adaptation, we believe that our findings will help alleviate such skepticism. To elaborate, if the elliptical profile described the Sxas’ profiles the best, then their tapered shape would still be a beneficial adaptation since the elliptical profile increases a column’s buckling strength by roughly 12% compared to its equivalent cylinder. However, the Sxa is best described by not just any beneficial taper, but by that for which the enhancement to buckling strength is the largest.

Finally, it is amazing to us that evolution has endowed such a simple animal—one that even lacks a brain—with a mechanical design that has engaged some of the most brilliant scientific minds^57^. We wonder whether the significance of the Sxa’s shape would have been identified if the Clausen profile were not already known. To that end, we also wonder whether the lack of suitable mechanics models for other mechanical designs obscure their significance and thereby allow them to hide in plain sight.

## Methods

### SEM imaging of strongyloxea

Strongyloxea from *T. aurantia* sponges were received dried and separated from the surrounding spongin. The Sxa were first examined using a polarized light microscope (Nikon Ci Pol). Intact, undamaged Sxa were mounted to aluminum stubs using conductive carbon tape. The mounted Sxa were sputter coated with approximately 10 nm of carbon and then imaged with a scanning electron microscope (FEI Helios, or LEO 1530 VP) at roughly 500X magnification. At this magnification, the field of view was roughly 250 *μ*m × 200 *μ*m in the FEI Helios (130 *μ*m × 90 *μ*m in the LEO 1530 VP). Therefore, a complete image of a Sxa consisted of 7–14 overlapping frames. These frames were aligned and stitched together to make a single composite image using a Fourier transform-based phase correlation method implemented in ImageJ^58^. A representative composite image is shown in Supplementary Fig. S1.

### Extracting strongyloxea boundary geometry from SEM images

Each composite image was first converted to a binary image in which the Sxa and background are made up of white and black pixels, respectively. Points on the boundary of the Sxa were identified using the Moore-Neighbor tracing algorithm implemented in MATLAB’s Image Processing Toolbox^59^ (see Supplementary Fig. S1). There were roughly 15,000 boundary points obtained for each Sxa. A line was fit to these points to determine the orientation of each Sxa’s axis. We used this line as the axial—*z*—direction in the (*z, r*) coordinate system shown in Fig. 1(c) and Supplementary Fig. S1. The locations of the boundary points were translated so that the point with the smallest *z* coordinate was located at the origin. Finally, the locations of the boundary points were converted from pixels to micrometers using a scale bar taken from the first frame of each composite image.

### Denoising and subsampling strongyloxea boundary data

We divided a Sxa’s boundary points into 50 partitions so that the *z* coordinates of the points in the *j*^*th*^ partition satisfy (*j* − 1)*L*_*m*_/50 ≤ *z* ≤ *jL*_*m*_/50, where *j* = 1, …, 50 and *L*_*m*_ is the maximum *z* value of the boundary points. The average *z* and *r* coordinates of the points in each partition were interpolated along with the end points, (0, 0) and (*L*_*m*_, 0), to generate the Sxa’s midline (see Supplementary Fig. S1). We used the midline to divide the boundary points into two halves. Boundary points whose *r* coordinates were greater (resp. less) than those of the midline at the same *z* value constitute the upper (resp. lower) half-boundary. Each half-boundary was denoised using a Savitzky-Golay filter with a kernel size of approximately 1/12^*th*^ the total number of boundary points. The two sets, 
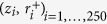
 and 
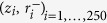
, were obtained by sampling the denoised upper and lower half-boundaries, respectively. The *z* values of these points were selected such that *z*_1_ = 0, *z*_250_ = *L*_*m*_ and 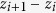, 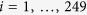 is a constant—i.e., the points are equally spaced in the *z* direction. The sets 
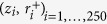
 and 
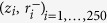
 constitute our model for a Sxa’s boundary and are used in Supplementary Section *Quantification of a Sxa*’*s axial and lateral symmetries* for quantifying the Sxa’s symmetries. After quantifying a Sxa’s symmetries, the 
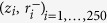
 set is discarded and the 
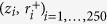
 set is used in the calculations and analysis in Sections *Measurement of Sxa profiles, Comparison with the Clausen profile*, and *Direct estimates of the Sxas*’ *buckling strengths*. In those sections we refer to the set 
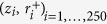
 as a Sxa’s profile and denote it as 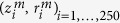.

## Additional Information

**How to cite this article**: Monn, M. A. and Kesari, H. A new structure-property connection in the skeletal elements of the marine sponge *Tethya aurantia* that guards against buckling instability. *Sci. Rep.* **7**, 39547; doi: 10.1038/srep39547 (2017).

**Publisher's note:** Springer Nature remains neutral with regard to jurisdictional claims in published maps and institutional affiliations.

## Supplementary Material

Supplementary Information

## Figures and Tables

**Table 1:** *mSSR* of the candidate profiles (*N* = 31).

	*mSSR* × 1000		
	Median	Mean	s.d.
Clausen, (4)–(5)	0.157	0.156	0.077
semiellipse, (S8)	0.247	0.281	0.165
triangle, (S9)	2.078	2.125	0.839
constant, (S10)	0.721	0.769	0.404
