# Supplementary material for: A new structure-property connection in the skeletal elements of the marine sponge Tethya aurantia that guards against buckling instability
Source: Sci Rep. 2017 Jan 4;7:39547. doi: 10.1038/srep39547 (PMC5209657; doi:10.1038/srep39547)
Supplement: Supplementary Information [file srep39547-s1.pdf]

Manuscript title: A new structure-property connection in the skeletal elements of the marine sponge *Tethya aurantia* that guards against buckling instability

Authors: Michael A. Monn, Haneesh Kesari

Quantification of a Sxa's axial and lateral symmetries

The majority of the Sxa we observed were straight, radially symmetric about their axis and symmetric across their lateral plane (see Supplementary Fig. S2 (a)). For this reason we idealized the Sxa as straight, tapered columns with circular cross-sections in our structural mechanics model (see Section *The structural mechanics model for the Sxa*).

However, approximately 34% of the 47 Sxa we imaged did not share the highly symmetric characteristics of the majority. For example, the axes of some Sxa were curved (e.g., see Supplementary Fig. S2 (d)). These asymmetric Sxa could be accidental deviations from the Sxa's body plan, or could support functions different from the stiffening function that we consider in this paper. Therefore, we chose not to compare the asymmetric Sxa with the profiles in Section *Comparison with the Clausen profile*. We characterized a Sxa as being asymmetric or not using the following procedure.

If a Sxa is straight and radially symmetric about its axis, then it also possesses a mirror symmetry across its transverse plane (see Supplementary Fig. S2 (a)). The metric M_B, defined in equation (S1), gives a measure of the Sxa's mirror symmetry across the transverse plane.

$$M_B(r_i^+, r_i^-) := 1 - \sqrt{\frac{\sum_{i=1}^{250}\left[|r_i^+| - |r_i^-|\right]^2}{\sum_{i=1}^{250}\left[(r_i^+)^2 + (r_i^-)^2\right]}}. \tag{S1}$$

Similarly, we define a metric M_A in equation (S2) that provides a measure of the Sxa's mirror symmetry across its lateral plane.

$$M_A(r_i^+, r_i^-) := 1 - \sqrt{\frac{\sum_{i=1}^{125}\left[(r_i^+ - r_{251-i}^+)^2 + (r_i^- - r_{251-i}^-)^2\right]}{\sum_{i=1}^{250}\left[(r_i^+)^2 + (r_i^-)^2\right]}} \tag{S2}$$

The M_A and M_B values lie between 0 and 1. When M_A (resp. M_B) equals unity then there is perfect mirror symmetry across the lateral (resp. transverse) plane. Several examples of shapes with different M_A and M_B values are shown in Supplementary Fig. S2 (b) and the M_A and M_B for the 47 Sxa are shown in Supplementary Fig. S2 (c). If a Sxa's M_A and M_B are both above certain values then we consider it to be symmetric, and consequently measure it and compare it to the profiles in Supplementary Section *Details of Sxa profile measurements* and Section *Comparison with the Clausen profile*. Otherwise, we categorize it as asymmetric and ignore it in all further analysis. We chose the cutoff values for M_A and M_B to be 0.85 and 0.84, respectively. Using this procedure we categorized 16 of the 47 Sxa that we imaged as being asymmetric.

Details of Sxa profile measurements

We define a Sxa's length $L_m = \max_i z_i^m$ and its maximum radius $R_m = \max_i r_i^m$, where $i = 1, \ldots, 250$. The mean values of R_m and L_m are 18.3 μm and 1.92 mm with standard deviations of 3.0 μm and 0.24 mm, respectively. From a Sxa's L_m and R_m values we compute its aspect ratio $\alpha_m = L_m/2R_m$. The mean and standard deviation of α_m for the measured Sxa are 53.6 and 8.7, respectively.

Deflection of a tapered beam in three-point bending predicted by Euler-Bernoulli theory

Consider a beam suspended across a trench of span L_t. The beam is simply supported at the trench edges and subjected a point force of magnitude F acting perpendicular to its axis at midspan—i.e., at $x = L_t/2$

(see Supplementary Fig. S3). The deformed shape of the beam is described in terms of the transverse deflection of its axis, $w(x)$. We denote w at midspan as w_0.

The beam behaves in a linear elastic fashion, and its elastic modulus is constant along its length. Because the beam's properties are linear elastic, its w_0–F response is linear and has a slope k.

The beam's second moment of area, I, can vary along its length, but we assume that the variation is symmetric across the midspan. Consequently, w will also be symmetric across the midspan. This allows us to consider only the half of the beam for which $0 \leq x \leq L_t/2$ for calculating w. From Euler-Bernoulli beam theory, w is governed by the ordinary differential equation

$$\frac{d^2w}{d\xi^2} = \frac{-24F}{k}\frac{\xi}{\eta(\xi)}, \tag{S3}$$

where $\xi = x/L_t \in (0,1/2)$ is the dimensionless coordinate in the x direction and $\eta(\xi) = I(L_t\xi)/I(L_t/2)$. The boundary conditions for the half-beam are

$$w(\xi)|_{\xi=0} = 0, \tag{S4}$$

$$\frac{dw(\xi)}{d\xi}|_{\xi=1/2} = 0, \tag{S5}$$

where equation (S5) comes as a consequence of the symmetry of w across the midspan.

For each Sxa we numerically compute w from equation (S3) subject to equations (S4)–(S5) and compare it with the deflections we measured from micrographs taken during the bending test. We find that the measurements and theoretical predictions match very well for 27 of the 30 Sxa that we tested. For a representative comparison, see Fig. 2 (d).

To compute w, we take L_t to be 1.278 mm, which is the distance between trench edges in our flexural testing device. For each Sxa, we measured F and obtained k by fitting a line to the w_0–F data. Since the micrographs taken during the bending tests are low magnification, we could not obtain detailed enough information from them to compute $\eta(\xi)$. Instead, we computed $\eta(\xi)$ using the profile of a randomly chosen Sxa that we measured in Section *Measurement of Sxa profiles*. We used the same $\eta(\xi)$ for each Sxa that we tested. We found that the w we computed from equation (S3) was relatively insensitive to which profile from Section *Measurement of Sxa profiles* we used.

Estimation of the distance between adjacent Sxa in a bundle

The arrangement of Sxa within a bundle is not well-characterized and is difficult to measure. In order to estimate the distance between neighboring Sxa in a bundle, we assume that they are evenly distributed within the bundle's cross-section. That is, they do not clump together in some regions of the cross-section and leave large expanses of spongin in others.

We represent a bundle's cross-section as a circular region with a radius $R_b = 177.5$ μm, which is half the mean thickness of a Sxa bundle.[1] We model the Sxa in this cross-section as N_s smaller circles all having a radius of $R_s = 18.3$ μm, which is the mean R_m from Supplementary Section *Details of Sxa profile measurements*. A previous study found that the cross-section of a Sxa bundle from a closely related species (*Tethya minutia*) contains anywhere from 10 to over 100 Sxa.[2] From these measurements we take an approximate average value and set $N_s = 50$.

To find what constitutes an evenly distributed arrangement of Sxa within a bundle, we treat the N_s smaller circles as if each has a positive electrostatic charge. Consequently, each circle exerts a repulsive force on every other circle and the magnitude of this force is inversely proportional to the square of the distance between them.

We describe the positions $(x_i^s, y_i^s)_{i=1...N_s}$ of the centers of the smaller circles using a cartesian coordinate system whose origin lies at the center of the circle representing the bundle's cross-section. We write the

potential energy $q(i,j)$ of the ith circle due to the presence of the jth circle as

$$q(i,j) = \begin{cases} C[h(i,j) - 2R_s]^{-1}, & h(i,j) > 2R_s, \\ \infty, & h(i,j) \leq 2R_s, \end{cases}$$

where $h(i,j) = [(x_i^s - x_j^s)^2 + (y_i^s - y_j^s)^2]^{1/2}$ is the distance between the two circles' centers, and C is a constant. We set $q(i,j) = \infty$ when the distance between the circles is less than $2R_s$ since two Sxa cannot occupy the same points in space. The total potential energy of the system is then given by

$$Q = \sum_{i=1}^{N_s-1} \sum_{j=i+1}^{N_s} q(i,j).$$

Without loss of generality we choose $C = 1$ and vary the positions of the circles $(x_i^s, y_i^s)_{i=1\ldots N_s}$ to minimize Q, subject to the constraint that $(x_i^s)^2 + (y_i^s)^2 \leq (R_b - R_s)^2$ for $i = 1\ldots N_s$. We minimize Q numerically for 50 random initial guesses of $(x_i^s, y_i^s)_{i=1\ldots N_s}$. We take the configuration of the circles corresponding to the smallest Q and compute the distance between each circle's center and the center of its nearest neighbor. The mean nearest neighbor distance in this configuration is 45.2 μm, which we use as the diameter of our RoC in Section *Computational mechanics calculations*.

Computational mechanics model of a Sxa in its RoC

We model the Sxa in its RoC as a rigid inclusion embedded in an elastic cylinder (see Supplementary Fig. S4 (a)). Since our goal is to determine the qualitative nature of the traction distribution on the Sxa at the initial stages, i.e., prior to the Sxa undergoing an significant motion, we assume that our computational mechanics model is axisymmetric. This assumption is expected to be true for the Sxa not lying on the surface of a bundle. Similarly, during the initial stages, the applied loads and deformation are likely to be symmetric across the lateral plane (see Supplementary Fig. S2 (a)). Therefore, we only consider half of the inclusion-cylinder pair in the computational mechanics calculations (see Supplementary Fig. S4 (a)). Ideally, we would like to model the exact shape of a Sxa. However among all models we considered, we found that the Clausen profile describes the Sxa's shape the best. Therefore, we represent the shape of the inclusion, $r(z)$, using a Clausen profile whose length and aspect ratio are mean(L_m) and mean(α_m), respectively. The length and diameter of the cylinder representing the RoC are 1.25mean(L_m) and 45 μm, respectively.

The different surfaces in our computational mechanics model are marked in Supplementary Fig. S4 (a). The inclusion and the cylinder are rigidly bonded along the surface Γ_1. Due to the lateral symmetry, we prescribe the displacements in the z direction on the surface Γ_4 to be zero. We also prescribe the displacements in the r direction on the surface Γ_3 to be zero as a way of modeling the fact that the matrix surrounding a Sxa is also rigidly bonded to neighboring Sxa at distances roughly equal to the RoC's diameter. We apply uniform tractions on the surface Γ_2 that are parallel to the Sxa's axis. The magnitude of the applied traction is not important since we only wish to understand the qualitative nature of the traction distribution on the Sxa during the initial stages.

We computed the axial component of the traction on Γ_1 from the Cauchy stress components, σ_{ij} for $i,j \in \{r,z\}$, as

$$t_z = \sigma_{zz} n_z + \sigma_{rz} n_r, \tag{S6}$$

where n_r and n_z are the radial and axial components of the unit vector normal to Γ_1. The components n_r and n_z can be computed from the inclusion's profile, $r(z)$, as

$$\begin{aligned} n_r &= (1 + r'^2)^{-1/2}, \\ n_z &= -r'(1 + r'^2)^{-1/2}, \end{aligned} \tag{S7}$$

where $r' = dr/dz$.

The net axial force transmitted across the Sxa's cross-section that is located at z' is $P_{\text{net}}(z') = \int_0^{z'} T_z(z)\,dz$, where

$$T_z(z) = 2\pi t_z(z) r(z)(1 + r'^2(z))^{1/2}$$

is the axial force per unit length acting on the Sxa. It can be seen, e.g., in Supplementary Fig. S4 (b), that T_z is highly localized at the Sxa's end. Specifically, we find that approximately 95% of the total transmitted axial force, $P = P_{\text{net}}(L_m/2)$, is found on the first 5% of the Sxa's length. The idealization of a point force of magnitude P acting on the Sxa's end would correspond to $T_z(z) = P\delta(z)$, where $\delta(\cdot)$ is the Dirac delta distribution. As can be seen in Supplementary Fig. S4 (b), the T_z distribution resembles a Dirac delta distribution.

We repeated this analysis using different Sxa profiles and various other types of boundary conditions. We found the results in all of the cases we considered to be qualitatively the same as the ones reported here.

Fitting profiles to the Sxas' shape

The dimensionless profile of a Sxa is given by the points, $(\zeta_i^m, \rho_i^m)_{i=1,\ldots,250} = (z_i^m/L_m, r_i^m/L_m)_{i=1,\ldots,250}$, from Section *Measurement of Sxa profiles*. We generate the Clausen profile points, $(\zeta_i, \rho_i)_{i=1,\ldots,250}$, so that $L = L_m$, $\zeta_i = \zeta_i^m$ and ρ_i satisfies equations (4)–(5) for each ζ_i. For each Sxa, we varied α in equation (4) to minimize the sum of squared residuals, $SSR = \sum_i (\rho_i^m - \rho_i)^2$, and we denote the minimum SSR as $mSSR$. To understand how well the Clausen profile describes the Sxa's taper, we also computed the $mSSR$ for three other profiles, which are given by,

$$\rho_{\ominus} = \alpha^{-1}\zeta^{1/2}(1-\zeta)^{1/2}, \tag{S8}$$

$$\rho_{\diamond} = \begin{cases} \alpha^{-1}\zeta, & 0 \le \zeta \le \frac{1}{2}, \\ \alpha^{-1}(1-\zeta), & \frac{1}{2} < \zeta \le 1, \end{cases} \tag{S9}$$

$$\rho_{\square} = (2\alpha)^{-1}, \tag{S10}$$

We found that the Clausen profile (equations (4)–(5)) has the lowest $mSSR$ for 25 of the 31 Sxa and the semiellipse profile (equation (S8)) has the lowest $mSSR$ for the remaining six.

Additional profile comparison using the Akaike information criterion

While the mean and median $mSSR$ is lowest for the Clausen profile, the semiellipse profile did have a lower $mSSR$ for approximately 19% of the Sxa. We clarify how much better the Clausen profile is compared to the semiellipse profile by finding the weight of evidence that the Clausen profile is the "best" of the candidate profiles. We consider the "best" profile for a particular Sxa to be the one that minimizes the Kullback-Liebler (K-L) distance. The K-L distance quantifies the amount information that is lost by using a model to approximate the true function from which the data was drawn.[3] Since this function is not known, the K-L distance cannot be computed directly. However, we can obtain an estimate of it by computing the Akaike Information Criterion (AIC).[3,4] For each Sxa, the fitted candidate profile with the lowest AIC value is also expected to be the K-L best profile.

We denote the $mSSR$ and AIC of the ith profile fitted to the jth Sxa as $mSSR_i^j$ and AIC_i^j, where $i \in \{\text{Clausen}, \text{semiellipse}, \text{triangle}, \text{constant}\}$ and $j = 1\ldots31$. The AIC_i^j is given by

$$AIC_i^j = N_j \log\left(\frac{mSSR_i^j}{N_j}\right) + 2(K_i + 2),$$

where N_j is the number of data points in the jth Sxa profile, and K_i is the number of free parameters in the ith model.[3] Here, N_j and K_i are both constants whose values are 250 and unity, respectively. From

the AIC_i^j values we can compute the relative likelihood that the ith profile is the the K-L best for the jth Sxa. This likelihood is referred to as the normalized Akaike weight, w_i^j, and is given by

$$w_i^j = \frac{e^{-\Delta_i^j/2}}{\sum_i e^{-\Delta_i^j/2}}, \tag{S11}$$

where $\Delta_i^j = AIC_i^j - \min_i AIC_i^j$.[3] The mean and standard deviation over j of w_i^j are given in Table S1. The ratio $mean_j(w^j_{\text{Clausen}}) : mean_j(w^j_{\text{semiellipse}})$ indicates that the Clausen profile is on average 4.55 times more likely to be the K-L best than the semiellipse profile.

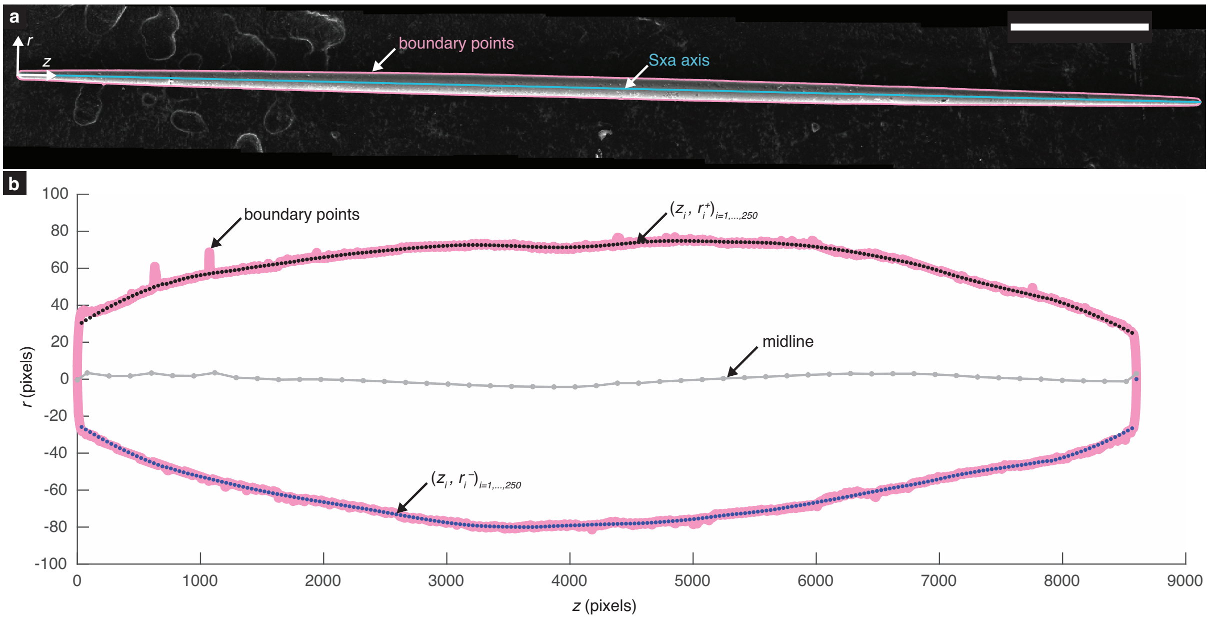

Figure S1: Sxa profile extracted from an SEM image. (a) A SEM image of a Sxa. The boundary points are shown in pink, and the Sxa's axis is shown in blue. The scale bar measures 250 μm. (b) The boundary points from (a) are shown in pink. These points are divided into two halves by the midline (gray), denoised by Savitsky-Golay filtering, and sampled to get the two sets of points $(z_i, r_i^+)_{i=1,\ldots,250}$ and $(z_i, r_i^-)_{i=1,\ldots,250}$ shown in black and blue, respectively.

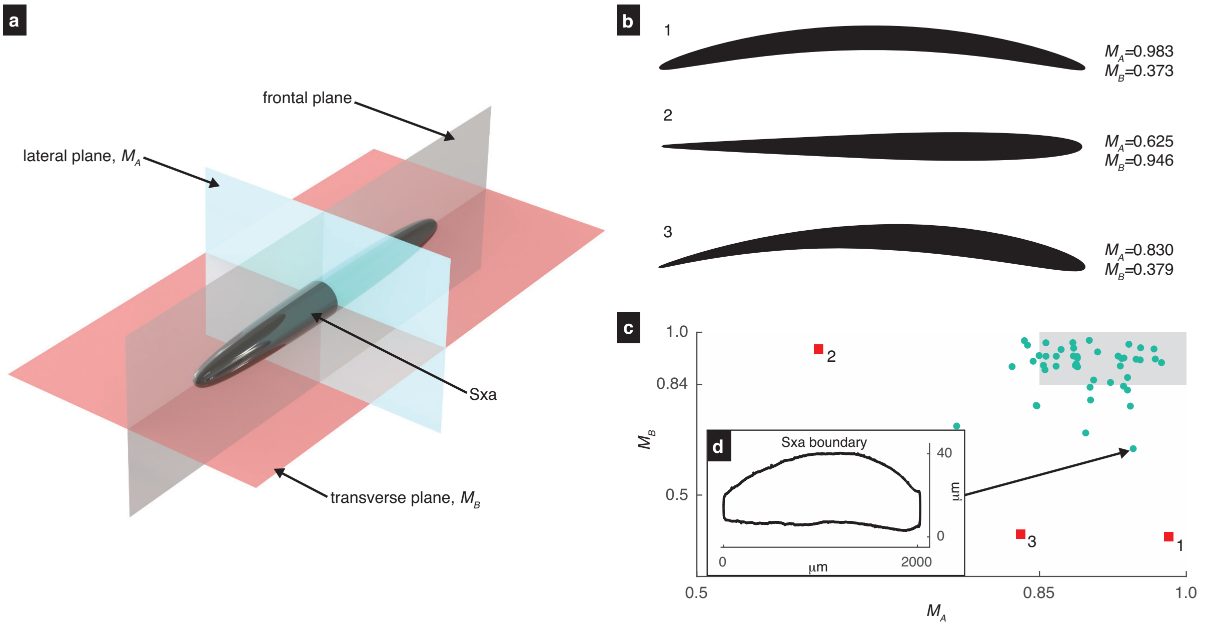

Figure S2: Axial and lateral symmetries of a Sxa. (a) Anatomical planes of a Sxa. Taking the frontal plane to be parallel to the imaging plane, we quantify a Sxa's symmetries across the transverse and lateral planes using the metrics M_B and M_A, respectively. (b) Three synthetically generated shapes with different M_A and M_B values. (c) the values of M_A and M_B for the 47 Sxa imaged, along with values from the three shapes in (b). The 31 Sxa whose (M_A, M_B) values lie inside the shaded region were used for measurement and comparison to the candidate profiles. (d) The boundary of a Sxa whose M_A and M_B fall outside of the cutoff values and is categorized as asymmetric.

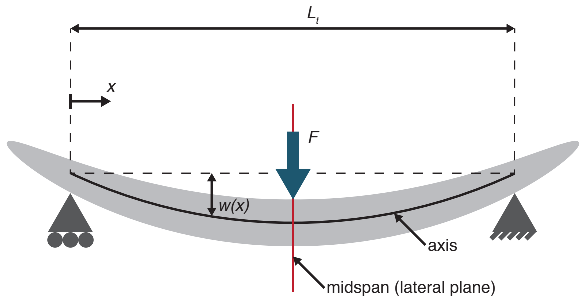

Figure S3: Bending of a beam with variable cross-section. A beam in a three-point bending configuration subjected to a transverse force of magnitude F at midspan. The beam's axis is indicated by a black line.

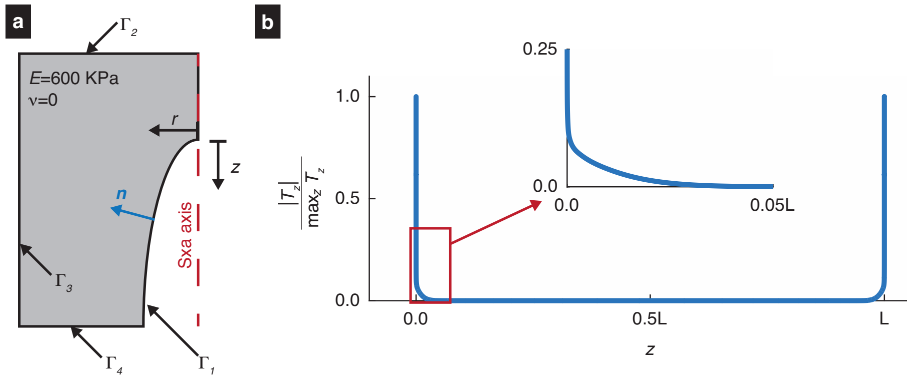

Figure S4: Computational mechanics model of a Sxa embedded in an elastic matrix. (a) Model geometry. The surface between the inclusion and the elastic cylinder is denoted by Γ_1. (b) Distribution of the axial force per unit length, T_z, along the Sxa's length. The inset shows a magnified view of the T_z distribution along the first 5% of the Sxa's length.

Table S1: Akaike weights, w, of the candidate profiles.

	mean	s.d.
Clausen, (4)–(5)	0.820 ±	0.374
semiellipse, (S8)	0.180 ±	0.374
triangle, (S9)	0.000 ±	0.000
constant, (S10)	0.000 ±	0.000

References

[1] Bavestrello, G., Calcinai, B., Ceccati, L., Cerrano, C. & Sarà, M. Skeletal development in two species of tethya (porifera, demospongiae). *Italian Journal of Zoology* **67**, 241–244 (2000).

[2] Nickel, M., Bullinger, E. & Beckmann, F. Functional morphology of tethya species (porifera): 2. three-dimensional morphometrics on spicules and skeleton superstructures of t. minuta. *Zoomorphology* **125**, 225–239 (2006).

[3] Burnham, K. & Anderson, D. Information and likelihood theory in *Model Selection and Multimodel Inference: a Practical Information-Theoretic Approach*, 49–84 (Springer Science & Business Media, 2002).

[4] Akaike, H. A new look at the statistical model identification. *Automatic Control, IEEE Transactions on* **19**, 716–723 (1974).
